# Recurrent Pleomorphic Adenoma of the Parotid Gland: Experience of 128 Patients with First Recurrence

**DOI:** 10.1155/2020/6645340

**Published:** 2020-12-23

**Authors:** Liyuan Dai, Weihua Lou, Qigen Fang, Xu Zhang

**Affiliations:** ^1^Department of Otorhinolaryngology Head and Neck Surgery, The First Affiliated Hospital of Zhengzhou University, Zhengzhou, China; ^2^Department of Head and Neck Thyroid, The Affiliated Cancer Hospital of Zhengzhou University, Henan Cancer Hospital, Zhengzhou, China

## Abstract

**Objective:**

Recurrence is common after inappropriate surgical procedures for parotid pleomorphic adenoma (PA). However, there are some controversies regarding intraoperative tumor rupture and disease recurrence; therefore, our goal was to clarify this relationship by describing our experience with 128 cases of recurrent parotid PA.

**Methods:**

Patients suffering from a first recurrence of parotid PA were prospectively enrolled, and data regarding the operation, pathology, immunohistochemistry, and recurrence pattern (outside the previous surgical field vs. inside the previous surgical field) were extracted and analyzed. The recurrent lesions were divided into two groups based on the location of nodularity.

**Results:**

Thirty-five patients had recurrent disease outside the previous surgical field; there were 105 nodules with a mean size of 1.0 (range: 0.4–3.0) cm and 983 nodules with a mean size of 1.55 (range: 0.5–4.5) cm within the field, and the difference was significant (*p*=0.001). The mean values of Ki-67 in nodules outside of and within the previous surgical field were 4.7% (range: 2%–10%) and 2.1% (range: 1%–7%), respectively, and the difference was significant (*p* < 0.001). In nodules outside the previous surgical field, cell-rich nodules were noted in 71.6% of cases; in nodules within the previous surgical field, cell-rich nodules were found in 30.4% of cases, and the difference was significant (*p* < 0.001).

**Conclusion:**

Tumor rupture is not the only cause of disease recurrence, and recurrent PAs outside the previous surgical field are smaller in size, have higher Ki-67 expression, and have more cell-rich nodules than those within the surgical scar.

## 1. Introduction

Pleomorphic adenoma (PA) is the most common benign tumor in the parotid gland, accounting for nearly 80%. Surgical management is the only suggested therapeutic strategy. The reported recurrence rate varies from 5% to 40%, adjusted to different surgical procedures [1–12]. Enucleation and total parotidectomy have the highest and lowest recurrence rates, respectively. Incomplete excision or violation of the tumor pseudosarcoma is the widely accepted cause contributing to disease recurrence [1–12]. Most cases of recurrent disease are located in the previous surgical field [3, 5, 6], but considerable numbers of recurrent tumors have occurred outside the parotid bed. The distant metastasis ability of PA by lymphatic or vascular vessels [1, 8] suggests that there might be additional explanations for cases of recurrence outside the parotid bed. Previous authors have questioned the relationship between tumor rupture and recurrence [13–15]; however, according to a retrospective study, it must be assumed that the data from medical records pertaining to tumor rupture during surgery are uncertain. Therefore, the current study aimed to describe our experience of treating 128 cases of recurrent PA with a focus on determining whether there were additional reasons for recurrence outside the previous surgical field.

## 2. Patients and Methods

The Zhengzhou University institutional research committee approved our study, and all participants signed an informed consent agreement. All methods were performed in accordance with the relevant guidelines and regulations.

From January 2012 to December 2017, 128 patients suffering from a first recurrence of parotid PA were prospectively enrolled. One hundred patients underwent a primary operation in another hospital, and information on the primary operation, including operative type and tumor rupture, was obtained from the previous operation records and the physician-in-charge. During the current admission, data regarding age, sex, operation, pathology, immunohistochemistry, postoperative complications, House–Brackmann scale, and follow-up were collected as clearly as possible.

All patients underwent preoperative ultrasound, CT, and/or MRI examinations to locate the recurrent disease as clearly as possible. During the operation, the recurrent lesion was first evaluated by at least two head and neck surgeons with at least 15 years of experience; then, the lesions were divided into two groups based on the location of nodularity. Group A consisted of nodularities located within the previous surgical scar field, and group B consisted of nodularities existing outside the previous surgical scar field (namely, located in the current fresh field). Nodules outside the previous surgical scar field were defined as nodules located in a fresh field at least 1 cm from the previous scar. Nodules were divided into three types based on different pathologic presentations: cell-rich, mucus-rich, and mixed nodules. Microvessel density (MVD) and Ki-67 were evaluated by immunohistochemistry, and MVD was calculated according to the positive staining of CD34 in vascular endothelial cells according to the method described by Weidner et al. [16].

Student's *t*-tests, chi-square tests, and nonparametric tests were used to analyze the differences between the two groups. Factors that were significant in univariate analysis were then analyzed in multivariate analysis to identify independent risk factors. All statistical analyses were performed using SPSS 20.0. A value of *p* < 0.05 was considered to be significant.

## 3. Results

Among the included 128 patients, there were 70 females and 58 males, the mean age was 48.1 (range: 16–83) years old, and the mean time between primary surgery and first recurrence was 5.4 (range: 0.5–12) years. Twenty-eight patients had undergone primary operations in our department with a mean age of 49.5 years; among them, there were 18 females and 10 males. One hundred patients had primary operations in other hospitals; there were 52 females and 48 males, with a mean age of 47.7 years. Before the current operation for recurrent disease, a total of 11 patients presented with facial palsy; among them, 7 patients complained of marginal mandibular branch and buccal branch paralysis, and 4 patients had marginal mandibular branch paralysis. The primary surgeries included 80 (62.5%) cases of enucleation, 33 (25.8%) cases of partial parotidectomy, and 15 (11.7%) cases of superficial parotidectomy. The difference regarding primary operation type and facial nerve paralysis between our hospital and others was both significant (*p* < 0.001 and *p*=0.014, respectively), and no apparent difference regarding other variables was noted (all *p* > 0.05) (Table 1).

During the current admission, all patients underwent extended total parotidectomy with preservation of the facial nerve and excision of the scar. In patients with preoperative normal facial nerve function, transient facial paralysis occurred in 90 (76.9%) patients, and permanent facial paralysis was seen in 10 (8.5%) patients (grade II: 6 cases; grade III: 4 cases).

A total of 30 (23.4%) patients had a history of tumor rupture during the first surgery, multiple nodules were reported in 100% of the 128 patients, and the association between tumor rupture and recurrence pattern was not significant (*p* > 0.05). The mean number of nodules found was 8.5 (range: 4–33), and the mean size of the nodules was 1.5 (range: 0.4–4.5) cm. Thirty-five (27.3%) patients had recurrent disease outside the previous surgical field; there were 105 nodules with a mean size of 1.0 (range: 0.4–3.0) cm and 983 nodules with a mean size of 1.55 (range: 0.5–4.5) cm within the field, and the difference regarding nodule size between the two groups was significant (*p*=0.001).

The mean values of Ki-67 in nodules outside of and within the previous surgical field were 4.7% (range: 2%–10%) and 2.1% (range: 1%–7%), respectively, and the difference was significant (*p* < 0.001). In nodules outside the previous surgical field, cell-rich nodules were noted in 71.6% of cases; in nodules within the previous surgical field, cell-rich nodules were found in 30.1% of cases, and the difference was significant (*p* < 0.001). The mean MVD was 6.2 (range: 4–13) and 5.9 (range: 3–12) in nodules outside of and within the previous surgical field, respectively, and the difference was not significant (*p* > 0.05).

Further multivariate analysis showed that the independence of Ki-67 index and cell-rich nodule was associated with the recurrence pattern (Table 2).

## 4. Discussion

Most previous authors speculated that the main reason for the recurrence of parotid PA was tumor rupture or tumor spill [1–12], but a few researchers questioned this viewpoint [13–15]. Navtig et al. [13] retrospectively reviewed the medical records of 346 patients with parotid PA; it was noted that 6 (2.5%) patients had recurrence, 26 patients had rupture of the capsule with macroscopic spillage of tumor cells, and recurrent tumors occurred in 2 of the patients. 87 patients had a surgical dissection close to the capsule, and 1 case developed recurrence. 3 cases of the remaining patients whose had surgical dissections without visualizing the tumor capsule developed recurrence. The 8% recurrence rate after capsule rupture was not significantly different from the 2% recurrence rate for the other patients. Moreover, there was no difference in the recurrence rate between patients with microscopic negative and positive surgical resection margins. In the paper published by Buchman et al. [14], intraoperative tumor spill or inadequate resection occurred in 17 cases with a salivary gland PA; the authors noted that inadequate resection, especially enucleation, rather than tumor spill was associated with local recurrence. Similarly, other researchers described that two (7.1%) of the 28 patients who had intraoperative tumor rupture had disease recurrence at a later stage. The rate was consistent with that for the rest of the patients. As many as 5 of the 9 primary tumors that subsequently recurred (56%) sent finger-like tumor extensions or pseudopodia outside the pseudocapsule. The occurrence of the structures was significantly more common than that of the examined uncomplicated cases (8%) and the tumors that ruptured during surgery (25%). In current study, it was noted that the frequency of facial nerve paralysis was lower in our hospital, and it might be partially contributed by our more meticulous nerve dissection and better surgical technique; additionally, even if there were significantly less primary operations of enucleation in our hospital, disease recurrence still could occur. This interesting finding of our study combined with others raised the debate that more underlying mechanisms might exist in addition to tumor rupture.

Approximately one-third to half of the recurrent nodules were located outside the previous surgical field [3–7], but these studies consisted of patients with different recurrence times, which is completely acceptable from the standpoint that the first recurrence is most likely related to primary surgery. The current study was the first to analyze possible additional reasons based on patients with first recurrence. It was noted that there was no significant association between tumor rupture and recurrence pattern (uninodular or multinodular), which was not surprising owing to the usual distant metastasis ability of PA [1, 7, 8]. Previous authors hypothesized that, in most cases, recurrent nodules start growing after spillage of epithelial tumor cells into the wound during surgery and followed by migration of small nests of tumor cells by vascular or lymphatic vessels that can then settle at a distance from the sites of origin [7]. In this way, it was interesting to discover whether there were some differences between nodules within and outside the surgical field. Unfortunately, few authors have analyzed this concern. Stennert et al. [4] first reported that cell-rich nodules mostly existed in the scar area, and in approximately 70% of the patients, complete absence of encapsulation could usually be noted. Similarly, we also noted that more cell-rich nodules existed outside the surgical field, and cell-rich nodules might tend to travel by lymphatic or vascular vessels. Moreover, the size of nodules outside the surgical field was significantly larger than that of nodules within the field, possibly because of the need for additional time for nodules outside the field to travel in vessels.

Immunohistochemistry analysis was one of the main goals in the current study. Ki-67 is usually used to describe proliferation ability, and the value is less than 5% in most parotid PAs; Ki-67 is also a potential indicator of recurrence and prognosis in salivary gland tumors [17, 18]. Interestingly, it was noted that there was a higher value of Ki-67 in nodules outside the field, and this finding might be associated with more cell-rich nodules indicating “distant metastasis” capacity. Similarly, Stennert et al. [7] reported that nodules with small cell-rich nodules had the highest proliferation index.

MVD was first introduced by Weidner et al. [16] and is usually analyzed in malignant tumors currently; MVD has been described to determine neoplastic neovascularization and is an independent prognostic factor associated with metastasis in many different cancers [19–21], and its value is commonly more than 30. Chen et al. [22] found that the MVD values in salivary adenoid cystic carcinoma and normal salivary gland tissues were 38.73 ± 8.96 and 11.15 ± 3.33, respectively. These values were statistically significant. Soares et al. [23] might have been the first to analyze the MVD in PAs, and the authors found that when comparing nonrecurrent with recurrent PA, although patients with recurrent PA presented with more aggressive clinical behavior than those with nonrecurrent PA, no significant difference was found in terms of lymphatic vessel density or MVD. In the current study, significant discrepancies among nodules inside or outside the previous surgical field were not observed. However, we thought that MVD might partially contribute to the existence of recurrent disease outside the surgical field. A larger sample size is needed to further study this question.

In summary, tumor rupture was often not associated with recurrence pattern; recurrent PA outside the surgical field was associated with a smaller tumor size, a higher Ki-67 index, and more cell-rich nodules than PA within the surgical scar.

## Figures and Tables

**Table 1:** Information of the included 128 patients.

Variables	Primary operation sites		Sum
	Our hospital (*n* = 28)	Others (*n* = 100)	
Age	49.5 (27–83)	47.7 (16–83)	48.1 (16–83)
Sex			
Female	18 (64.3%)	52 (52.0%)	70 (54.7%)
Male	10 (35.7%)	48 (48.0%)	58 (45.3%)
Primary operation type^*∗*^			
Enucleation	1 (3.6%)	79 (79.0%)	80 (62.5%)
Partial parotidectomy	15 (53.6%)	18 (18.0%)	33 (25.8%)
Superficial parotidectomy	12 (42.9%)	3 (3.0%)	15 (11.7%)
Facial nerve paralysis^#^			
Marginal mandibular branch	2 (7.1%)	2 (2.0%)	4 (3.1%)
Marginal mandibular and buccal branches	4 (14.3%)	3 (3.0%)	7 (5.5%)
Tumor rupture	5 (17.9%)	25 (25.0%)	30 (23.4%)

^*∗*^The difference regarding primary operation type between the two groups was significant (Fisher's exact test, *p* < 0.001). ^#^The difference regarding facial nerve paralysis between the two groups was significant (Fisher's exact test, *p*=0.014).

**Table 2:** Association between clinical pathologic variables and recurrence pattern in the 128 patients.

Variables	Univariate		Multivariate		
	Inside (*n* = 93)	Outside (*n* = 35)	*p*	*p*	Or [95%CI]
Age					
≥50	47 (50.5%)	20 (57.1%)	—	—	—
<50	46 (49.5%)	15 (42.9%)	0.505	—	—
Sex					
Male	42 (45.2%)	16 (45.7%)	—	—	—
Female	51 (54.8%)	19 (54.3%)	0.955	—	—
Tumor rupture					
Yes	20 (21.5%)	10 (28.6%)	—	—	—
No	73 (78.5%)	25 (71.4%)	0.400	—	—
Nodule size					
≥2.0 cm	29 (31.2%)	1 (2.9%)	—	—	—
<2.0 cm	64 (68.8%)	34 (97.1%)	0.001	0.125	5.415[0.875–18.632]
Recurrence time					
<5 years	38 (40.9%)	10 (28.6%)	—	—	—
≥5 years	55 (59.1%)	25 (71.4%)	0.201	—	—
Ki-67					
<5.0%	79 (84.9%)	19 (54.3%)	—	—	—
≥5.0%	14 (15.1%)	16 (45.7%)	<0.001	0.014	2.347[1.224–5.687]
Nodule type					
Others	65 (69.9%)	10 (28.6%)	—	—	—
Cell-rich	28 (30.1%)	25 (71.4%)	<0.001	0.015	2.067 [1.449–6.777]
MVD^#^					
≥6	40 (43.0%)	18 (51.4%)	—	—	—
<6	53 (57.0%)	17 (48.6%)	0.394	—	—
Primary operation					
Enucleation	56 (60.2%)	24 (68.6%)	—	—	—
Partial parotidectomy	25 (26.9%)	8 (22.9%)	—	—	—
Superficial parotidectomy	12 (12.9%)	3 (8.6%)	0.673	—	—

^#^MVD: microvessel density.

## Data Availability

All data generated or analyzed during this study are included in this published article. And the primary data could be obtained from the corresponding author upon request.
